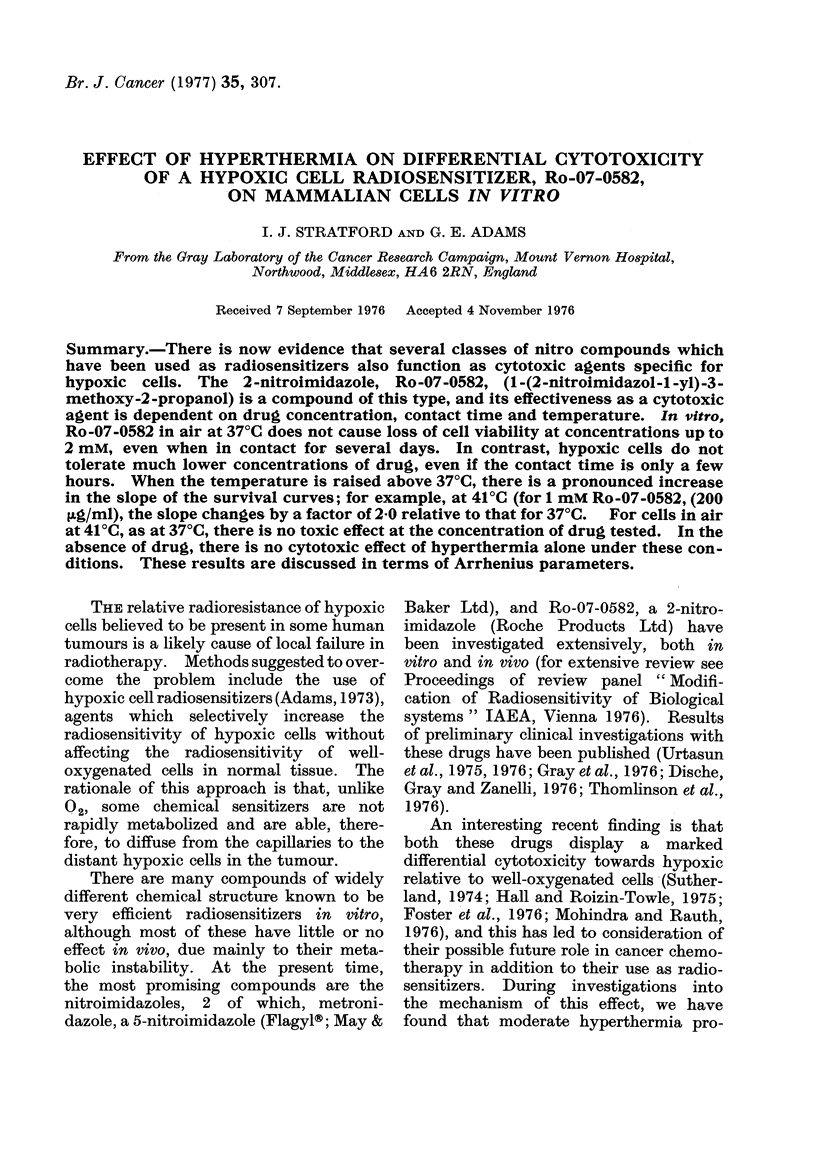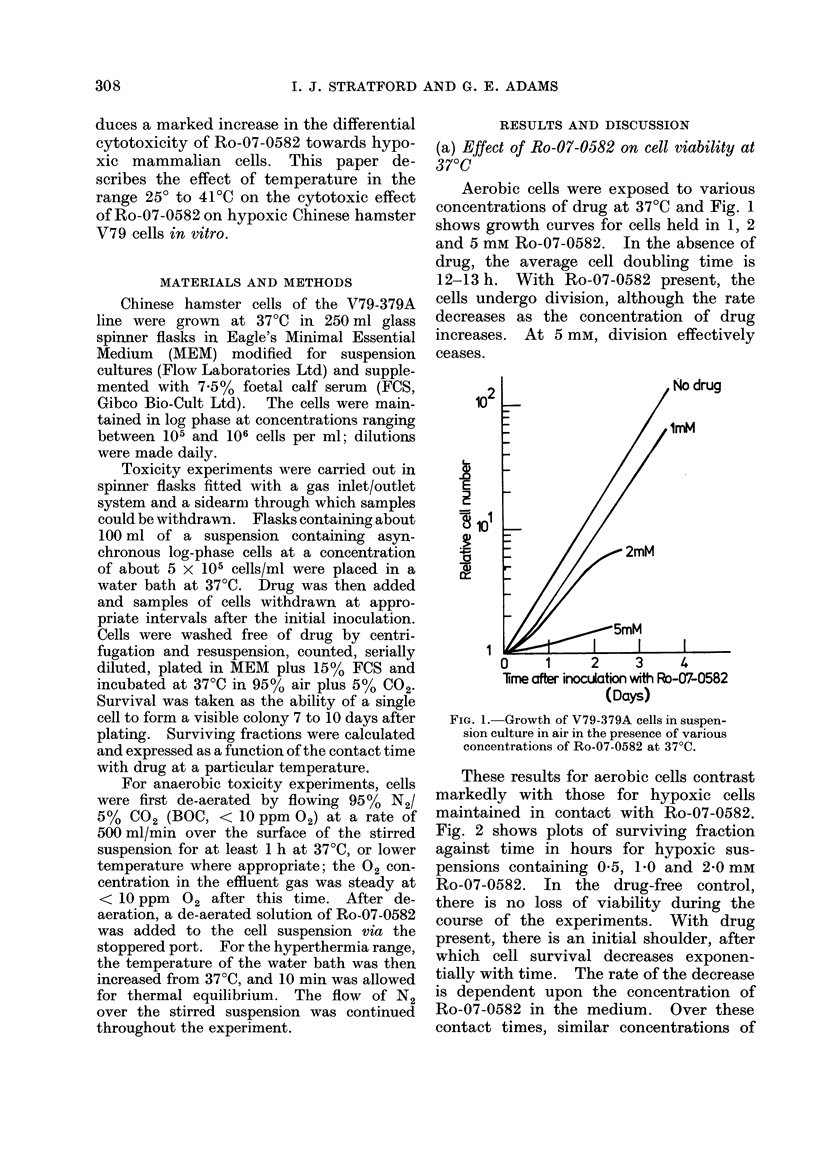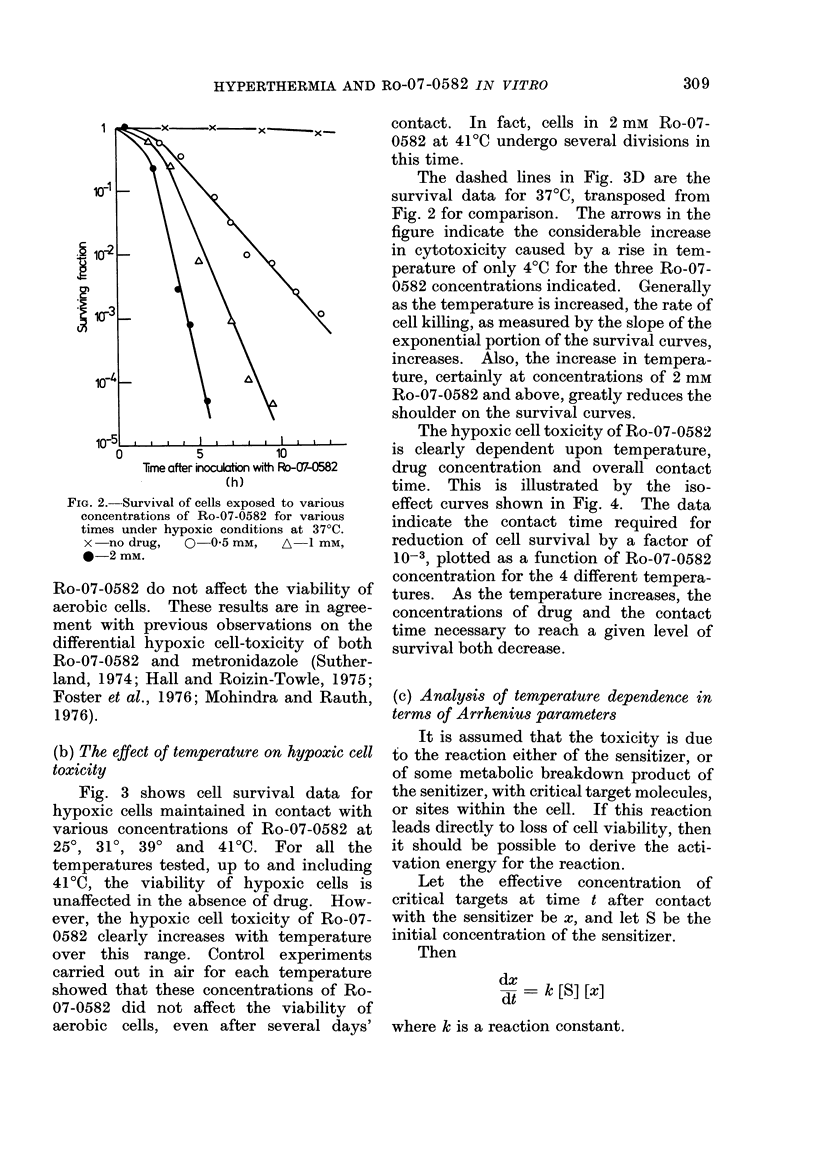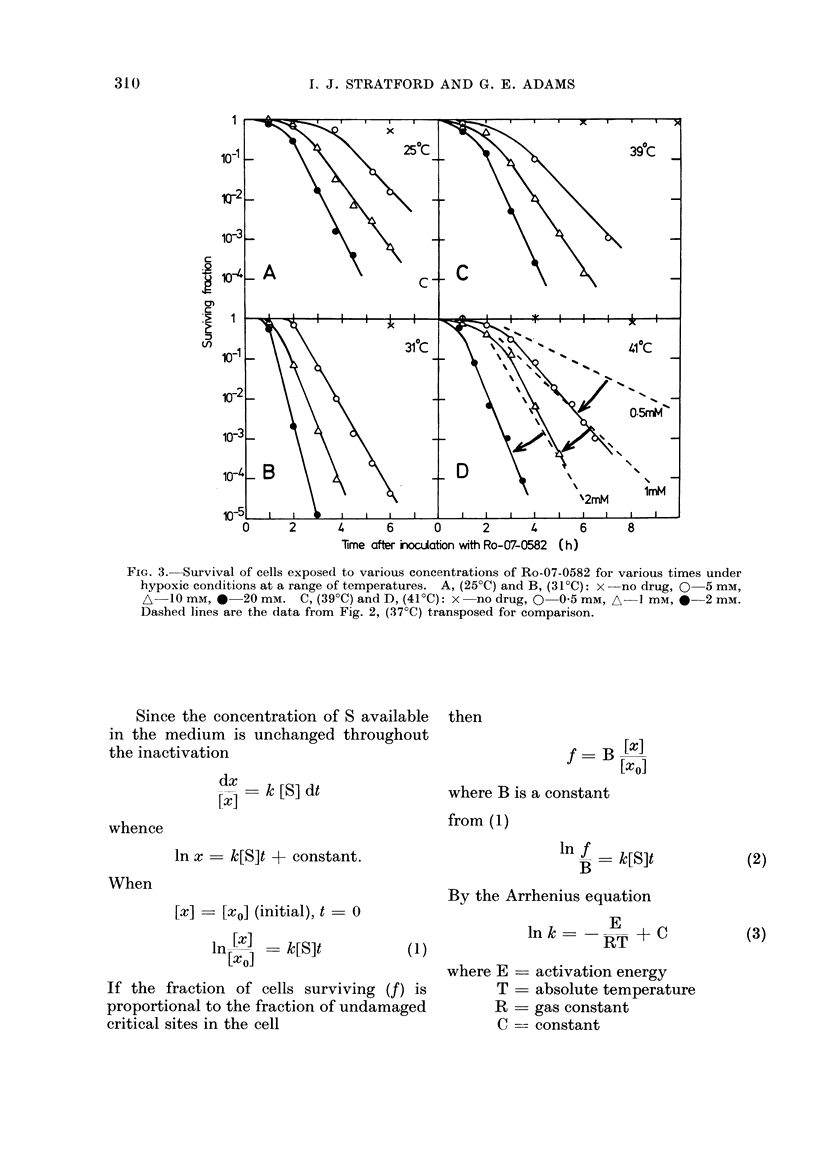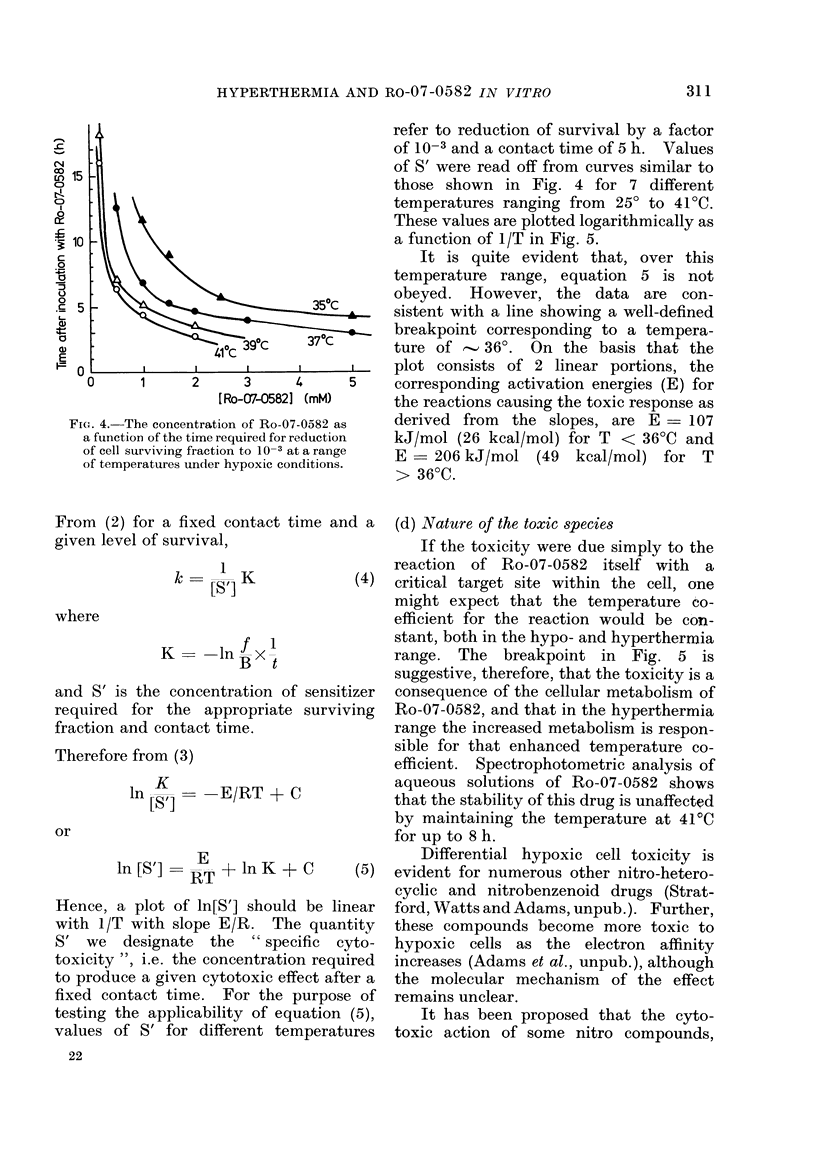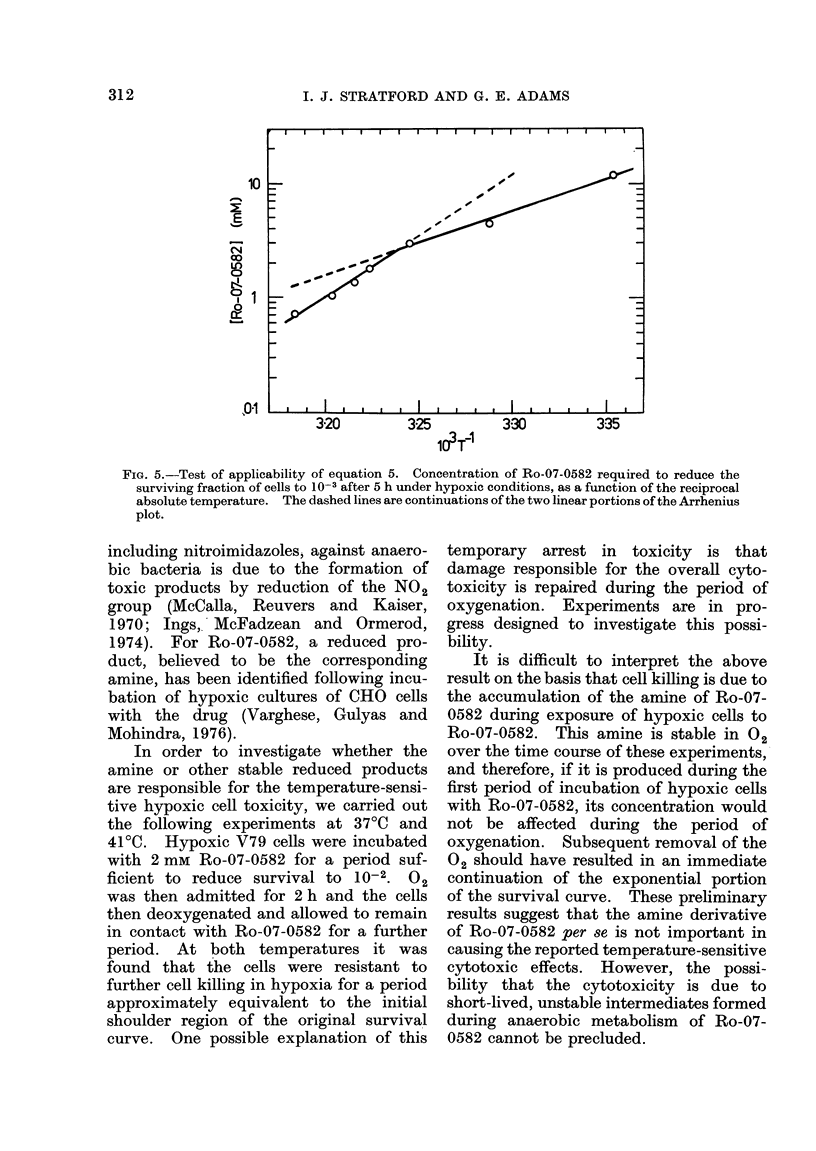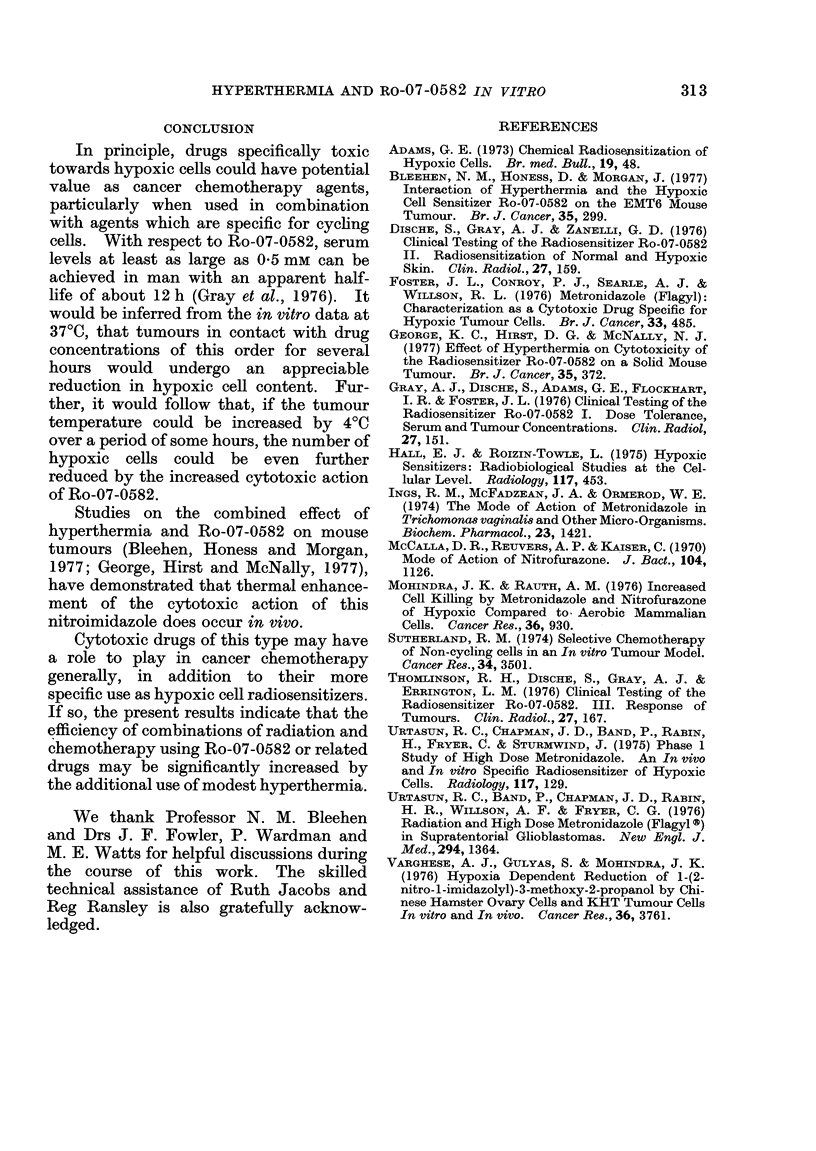# Effect of hyperthermia on differential cytotoxicity of a hypoxic cell radiosensitizer, Ro-07-0582, on mammalian cells in vitro.

**DOI:** 10.1038/bjc.1977.44

**Published:** 1977-03

**Authors:** I. J. Stratford, G. E. Adams

## Abstract

There is now evidence that several classes of nitro compounds which have been used as radiosensitizers also function as cytotoxic agents specific for hypoxic cells. The 2-nitroimidazole, Ro-07-0582, (1-(2-nitroimidazol-1-yl)-3-methoxy-2-propanol) is a compound of this type, and its effectiveness as a cytotoxic agent is dependent on drug concentration, contact time and temperature. In vitro, Ro-07-0582 in air at 37 degrees C does not cause loss of cell viability at concentrations up to 2 mM, even when in contact for several days. In contrast, hypoxic cells do not tolerate much lower concentrations of drugs, even if the contact time is only a few hours. When the temperature is raised above 37 degrees C, there is a pronounced increase in the slope of the survival curves; for example, at 41 degrees C (for 1 mM Ro-07-0582, (200 microng/ml), the slope changes by a factor of 2-0 relative to that for 37 degrees C. For cells in air at 41 degrees C, as at 37 degrees C, there is no toxic effect at the concentration of drug tested. In the absence of drug, there is no cytotoxic effect of hyperthermia alone under these conditions. These results are discussed in terms of Arrhenius parameters.


*Br. J. Cancer* (1977) **35**, 307.

### EFFECT OF HYPERTHERMIA ON DIFFERENTIAL CYTOTOXICITY OF A HYPOXIC CELL RADIOSENSITIZER, Ro-07-0582, ON MAMMALIAN CELLS *IN VITRO*

I. J. STRATFORD AND G. E. ADAMS

*From the Gray Laboratory of the Cancer Research Campaign, Mount Vernon Hospital, Northwood, Middlesex, HA6 2RN, England*

Received 7 September 1976 Accepted 4 November 1976

**Summary.—There is now evidence that several classes of nitro compounds which have been used as radiosensitizers also function as cytotoxic agents specific for hypoxic cells. The 2-nitroimidazole, Ro-07-0582, (1-(2-nitroimidazol-1-yl)-3-methoxy-2-propanol) is a compound of this type, and its effectiveness as a cytotoxic agent is dependent on drug concentration, contact time and temperature. *In vitro,* Ro-07-0582 in air at 37°C does not cause loss of cell viability at concentrations up to 2 mM, even when in contact for several days. In contrast, hypoxic cells do not tolerate much lower concentrations of drug, even if the contact time is only a few hours. When the temperature is raised above 37°C, there is a pronounced increase in the slope of the survival curves; for example, at 41°C (for 1 mM Ro-07-0582, (200 μg/ml), the slope changes by a factor of 2·0 relative to that for 37°C. For cells in air at 41°C, as at 37°C, there is no toxic effect at the concentration of drug tested. In the absence of drug, there is no cytotoxic effect of hyperthermia alone under these conditions. These results are discussed in terms of Arrhenius parameters.**

THE relative radioresistance of hypoxic cells believed to be present in some human tumours is a likely cause of local failure in radiotherapy. Methods suggested to overcome the problem include the use of hypoxic cell radiosensitizers (Adams, 1973), agents which selectively increase the radiosensitivity of hypoxic cells without affecting the radiosensitivity of well-oxygenated cells in normal tissue. The rationale of this approach is that, unlike $O_2$, some chemical sensitizers are not rapidly metabolized and are able, therefore, to diffuse from the capillaries to the distant hypoxic cells in the tumour.

There are many compounds of widely different chemical structure known to be very efficient radiosensitizers *in vitro*, although most of these have little or no effect *in vivo*, due mainly to their metabolic instability. At the present time, the most promising compounds are the nitroimidazoles, 2 of which, metronidazole, a 5-nitroimidazole (Flagyl®; May & Baker Ltd), and Ro-07-0582, a 2-nitroimidazole (Roche Products Ltd) have been investigated extensively, both *in vitro* and *in vivo* (for extensive review see Proceedings of review panel "Modification of Radiosensitivity of Biological systems" IAEA, Vienna 1976). Results of preliminary clinical investigations with these drugs have been published (Urtasun *et al.*, 1975, 1976; Gray *et al.*, 1976; Dische, Gray and Zanelli, 1976; Thomlinson *et al.*, 1976).

An interesting recent finding is that both these drugs display a marked differential cytotoxicity towards hypoxic relative to well-oxygenated cells (Sutherland, 1974; Hall and Roizin-Towle, 1975; Foster *et al.*, 1976; Mohindra and Rauth, 1976), and this has led to consideration of their possible future role in cancer chemotherapy in addition to their use as radiosensitizers. During investigations into the mechanism of this effect, we have found that moderate hyperthermia produces a marked increase in the differential cytotoxicity of Ro-07-0582 towards hypoxic mammalian cells. This paper describes the effect of temperature in the range 25° to 41°C on the cytotoxic effect of Ro-07-0582 on hypoxic Chinese hamster V79 cells *in vitro.*

### MATERIALS AND METHODS

Chinese hamster cells of the V79-379A line were grown at 37°C in 250 ml glass spinner flasks in Eagle's Minimal Essential Medium (MEM) modified for suspension cultures (Flow Laboratories Ltd) and supplemented with 7·5% foetal calf serum (FCS, Gibco Bio-Cult Ltd). The cells were maintained in log phase at concentrations ranging between $10^5$ and $10^6$ cells per ml; dilutions were made daily.

Toxicity experiments were carried out in spinner flasks fitted with a gas inlet/outlet system and a sidearm through which samples could be withdrawn. Flasks containing about 100 ml of a suspension containing asynchronous log-phase cells at a concentration of about $5 \times 10^5$ cells/ml were placed in a water bath at 37°C. Drug was then added and samples of cells withdrawn at appropriate intervals after the initial inoculation. Cells were washed free of drug by centrifugation and resuspension, counted, serially diluted, plated in MEM plus 15% FCS and incubated at 37°C in 95% air plus 5% $CO_2$. Survival was taken as the ability of a single cell to form a visible colony 7 to 10 days after plating. Surviving fractions were calculated and expressed as a function of the contact time with drug at a particular temperature.

For anaerobic toxicity experiments, cells were first de-aerated by flowing 95% $N_2$/5% $CO_2$ (BOC, $< 10$ ppm $O_2$) at a rate of 500 ml/min over the surface of the stirred suspension for at least 1 h at 37°C, or lower temperature where appropriate; the $O_2$ concentration in the effluent gas was steady at $< 10$ ppm $O_2$ after this time. After de-aeration, a de-aerated solution of Ro-07-0582 was added to the cell suspension *via* the stoppered port. For the hyperthermia range, the temperature of the water bath was then increased from 37°C, and 10 min was allowed for thermal equilibrium. The flow of $N_2$ over the stirred suspension was continued throughout the experiment.

### RESULTS AND DISCUSSION

### (a) *Effect of Ro-07-0582 on cell viability at 37°C*

Aerobic cells were exposed to various concentrations of drug at 37°C and Fig. 1 shows growth curves for cells held in 1, 2 and 5 mM Ro-07-0582. In the absence of drug, the average cell doubling time is 12–13 h. With Ro-07-0582 present, the cells undergo division, although the rate decreases as the concentration of drug increases. At 5 mM, division effectively ceases.

FIG. 1.—Growth of V79-379A cells in suspension culture in air in the presence of various concentrations of Ro-07-0582 at 37°C.

These results for aerobic cells contrast markedly with those for hypoxic cells maintained in contact with Ro-07-0582. Fig. 2 shows plots of surviving fraction against time in hours for hypoxic suspensions containing 0·5, 1·0 and 2·0 mM Ro-07-0582. In the drug-free control, there is no loss of viability during the course of the experiments. With drug present, there is an initial shoulder, after which cell survival decreases exponentially with time. The rate of the decrease is dependent upon the concentration of Ro-07-0582 in the medium. Over these contact times, similar concentrations of

FIG. 2.—Survival of cells exposed to various concentrations of Ro-07-0582 for various times under hypoxic conditions at 37°C. ×—no drug, ○—0·5 mM, △—1 mM, ●—2 mM.

Ro-07-0582 do not affect the viability of aerobic cells. These results are in agreement with previous observations on the differential hypoxic cell-toxicity of both Ro-07-0582 and metronidazole (Sutherland, 1974; Hall and Roizin-Towle, 1975; Foster *et al.*, 1976; Mohindra and Rauth, 1976).

(b) *The effect of temperature on hypoxic cell toxicity*

Fig. 3 shows cell survival data for hypoxic cells maintained in contact with various concentrations of Ro-07-0582 at 25°, 31°, 39° and 41°C. For all the temperatures tested, up to and including 41°C, the viability of hypoxic cells is unaffected in the absence of drug. However, the hypoxic cell toxicity of Ro-07-0582 clearly increases with temperature over this range. Control experiments carried out in air for each temperature showed that these concentrations of Ro-07-0582 did not affect the viability of aerobic cells, even after several days' contact. In fact, cells in 2 mM Ro-07-0582 at 41°C undergo several divisions in this time.

The dashed lines in Fig. 3D are the survival data for 37°C, transposed from Fig. 2 for comparison. The arrows in the figure indicate the considerable increase in cytotoxicity caused by a rise in temperature of only 4°C for the three Ro-07-0582 concentrations indicated. Generally as the temperature is increased, the rate of cell killing, as measured by the slope of the exponential portion of the survival curves, increases. Also, the increase in temperature, certainly at concentrations of 2 mM Ro-07-0582 and above, greatly reduces the shoulder on the survival curves.

The hypoxic cell toxicity of Ro-07-0582 is clearly dependent upon temperature, drug concentration and overall contact time. This is illustrated by the isoeffect curves shown in Fig. 4. The data indicate the contact time required for reduction of cell survival by a factor of $10^{-3}$, plotted as a function of Ro-07-0582 concentration for the 4 different temperatures. As the temperature increases, the concentrations of drug and the contact time necessary to reach a given level of survival both decrease.

(c) *Analysis of temperature dependence in terms of Arrhenius parameters*

It is assumed that the toxicity is due to the reaction either of the sensitizer, or of some metabolic breakdown product of the senitizer, with critical target molecules, or sites within the cell. If this reaction leads directly to loss of cell viability, then it should be possible to derive the activation energy for the reaction.

Let the effective concentration of critical targets at time $t$ after contact with the sensitizer be $x$, and let S be the initial concentration of the sensitizer.

Then

$$\frac{\mathrm{d}x}{\mathrm{d}t} = k\,[\mathrm{S}]\,[x]$$

where $k$ is a reaction constant.

FIG. 3.—Survival of cells exposed to various concentrations of Ro-07-0582 for various times under hypoxic conditions at a range of temperatures. A, (25°C) and B, (31°C): ×—no drug, ○—5 mM, △—10 mM, ●—20 mM. C, (39°C) and D, (41°C): ×—no drug, ○—0·5 mM, △—1 mM, ●—2 mM. Dashed lines are the data from Fig. 2, (37°C) transposed for comparison.

Since the concentration of S available in the medium is unchanged throughout the inactivation

$$\frac{dx}{[x]} = k\,[S]\,dt$$

whence

$$\ln x = k[S]t + \text{constant}.$$

When

$$[x] = [x_0] \text{ (initial)}, t = 0$$

$$\ln\frac{[x]}{[x_0]} = k[S]t \qquad (1)$$

If the fraction of cells surviving ($f$) is proportional to the fraction of undamaged critical sites in the cell then

$$f = B\frac{[x]}{[x_0]}$$

where B is a constant from (1)

$$\ln\frac{f}{B} = k[S]t \qquad (2)$$

By the Arrhenius equation

$$\ln k = -\frac{E}{RT} + C \qquad (3)$$

where E = activation energy
T = absolute temperature
R = gas constant
C = constant

FIG. 4.—The concentration of Ro-07-0582 as a function of the time required for reduction of cell surviving fraction to $10^{-3}$ at a range of temperatures under hypoxic conditions.

From (2) for a fixed contact time and a given level of survival,

$$k = \frac{1}{[S']} K \qquad (4)$$

where

$$K = -\ln \frac{f}{B} \times \frac{1}{t}$$

and S′ is the concentration of sensitizer required for the appropriate surviving fraction and contact time.

Therefore from (3)

$$\ln \frac{K}{[S']} = -E/RT + C$$

or

$$\ln [S'] = \frac{E}{RT} + \ln K + C \qquad (5)$$

Hence, a plot of ln[S′] should be linear with 1/T with slope E/R. The quantity S′ we designate the "specific cytotoxicity", i.e. the concentration required to produce a given cytotoxic effect after a fixed contact time. For the purpose of testing the applicability of equation (5), values of S′ for different temperatures refer to reduction of survival by a factor of $10^{-3}$ and a contact time of 5 h. Values of S′ were read off from curves similar to those shown in Fig. 4 for 7 different temperatures ranging from 25° to 41°C. These values are plotted logarithmically as a function of 1/T in Fig. 5.

It is quite evident that, over this temperature range, equation 5 is not obeyed. However, the data are consistent with a line showing a well-defined breakpoint corresponding to a temperature of ~ 36°. On the basis that the plot consists of 2 linear portions, the corresponding activation energies (E) for the reactions causing the toxic response as derived from the slopes, are E = 107 kJ/mol (26 kcal/mol) for T < 36°C and E = 206 kJ/mol (49 kcal/mol) for T > 36°C.

(d) *Nature of the toxic species*

If the toxicity were due simply to the reaction of Ro-07-0582 itself with a critical target site within the cell, one might expect that the temperature coefficient for the reaction would be constant, both in the hypo- and hyperthermia range. The breakpoint in Fig. 5 is suggestive, therefore, that the toxicity is a consequence of the cellular metabolism of Ro-07-0582, and that in the hyperthermia range the increased metabolism is responsible for that enhanced temperature coefficient. Spectrophotometric analysis of aqueous solutions of Ro-07-0582 shows that the stability of this drug is unaffected by maintaining the temperature at 41°C for up to 8 h.

Differential hypoxic cell toxicity is evident for numerous other nitro-heterocyclic and nitrobenzenoid drugs (Stratford, Watts and Adams, unpub.). Further, these compounds become more toxic to hypoxic cells as the electron affinity increases (Adams *et al.*, unpub.), although the molecular mechanism of the effect remains unclear.

It has been proposed that the cytotoxic action of some nitro compounds,

FIG. 5.—Test of applicability of equation 5. Concentration of Ro-07-0582 required to reduce the surviving fraction of cells to $10^{-3}$ after 5 h under hypoxic conditions, as a function of the reciprocal absolute temperature. The dashed lines are continuations of the two linear portions of the Arrhenius plot.

including nitroimidazoles, against anaerobic bacteria is due to the formation of toxic products by reduction of the $NO_2$ group (McCalla, Reuvers and Kaiser, 1970; Ings, McFadzean and Ormerod, 1974). For Ro-07-0582, a reduced product, believed to be the corresponding amine, has been identified following incubation of hypoxic cultures of CHO cells with the drug (Varghese, Gulyas and Mohindra, 1976).

In order to investigate whether the amine or other stable reduced products are responsible for the temperature-sensitive hypoxic cell toxicity, we carried out the following experiments at 37°C and 41°C. Hypoxic V79 cells were incubated with 2 mM Ro-07-0582 for a period sufficient to reduce survival to $10^{-2}$. $O_2$ was then admitted for 2 h and the cells then deoxygenated and allowed to remain in contact with Ro-07-0582 for a further period. At both temperatures it was found that the cells were resistant to further cell killing in hypoxia for a period approximately equivalent to the initial shoulder region of the original survival curve. One possible explanation of this temporary arrest in toxicity is that damage responsible for the overall cytotoxicity is repaired during the period of oxygenation. Experiments are in progress designed to investigate this possibility.

It is difficult to interpret the above result on the basis that cell killing is due to the accumulation of the amine of Ro-07-0582 during exposure of hypoxic cells to Ro-07-0582. This amine is stable in $O_2$ over the time course of these experiments, and therefore, if it is produced during the first period of incubation of hypoxic cells with Ro-07-0582, its concentration would not be affected during the period of oxygenation. Subsequent removal of the $O_2$ should have resulted in an immediate continuation of the exponential portion of the survival curve. These preliminary results suggest that the amine derivative of Ro-07-0582 *per se* is not important in causing the reported temperature-sensitive cytotoxic effects. However, the possibility that the cytotoxicity is due to short-lived, unstable intermediates formed during anaerobic metabolism of Ro-07-0582 cannot be precluded.

### CONCLUSION

In principle, drugs specifically toxic towards hypoxic cells could have potential value as cancer chemotherapy agents, particularly when used in combination with agents which are specific for cycling cells. With respect to Ro-07-0582, serum levels at least as large as 0·5 mM can be achieved in man with an apparent half-life of about 12 h (Gray *et al.*, 1976). It would be inferred from the *in vitro* data at 37°C, that tumours in contact with drug concentrations of this order for several hours would undergo an appreciable reduction in hypoxic cell content. Further, it would follow that, if the tumour temperature could be increased by 4°C over a period of some hours, the number of hypoxic cells could be even further reduced by the increased cytotoxic action of Ro-07-0582.

Studies on the combined effect of hyperthermia and Ro-07-0582 on mouse tumours (Bleehen, Honess and Morgan, 1977; George, Hirst and McNally, 1977), have demonstrated that thermal enhancement of the cytotoxic action of this nitroimidazole does occur *in vivo*.

Cytotoxic drugs of this type may have a role to play in cancer chemotherapy generally, in addition to their more specific use as hypoxic cell radiosensitizers. If so, the present results indicate that the efficiency of combinations of radiation and chemotherapy using Ro-07-0582 or related drugs may be significantly increased by the additional use of modest hyperthermia.

We thank Professor N. M. Bleehen and Drs J. F. Fowler, P. Wardman and M. E. Watts for helpful discussions during the course of this work. The skilled technical assistance of Ruth Jacobs and Reg Ransley is also gratefully acknowledged.

### REFERENCES

ADAMS, G. E. (1973) Chemical Radiosensitization of Hypoxic Cells. *Br. med. Bull.*, **19**, 48.

BLEEHEN, N. M., HONESS, D. & MORGAN, J. (1977) Interaction of Hyperthermia and the Hypoxic Cell Sensitizer Ro-07-0582 on the EMT6 Mouse Tumour. *Br. J. Cancer*, **35**, 299.

DISCHE, S., GRAY, A. J. & ZANELLI, G. D. (1976) Clinical Testing of the Radiosensitizer Ro-07-0582 II. Radiosensitization of Normal and Hypoxic Skin. *Clin. Radiol.*, **27**, 159.

FOSTER, J. L., CONROY, P. J., SEARLE, A. J. & WILLSON, R. L. (1976) Metronidazole (Flagyl): Characterization as a Cytotoxic Drug Specific for Hypoxic Tumour Cells. *Br. J. Cancer*, **33**, 485.

GEORGE, K. C., HIRST, D. G. & MCNALLY, N. J. (1977) Effect of Hyperthermia on Cytotoxicity of the Radiosensitizer Ro-07-0582 on a Solid Mouse Tumour. *Br. J. Cancer*, **35**, 372.

GRAY, A. J., DISCHE, S., ADAMS, G. E., FLOCKHART, I. R. & FOSTER, J. L. (1976) Clinical Testing of the Radiosensitizer Ro-07-0582 I. Dose Tolerance, Serum and Tumour Concentrations. *Clin. Radiol*, **27**, 151.

HALL, E. J. & ROIZIN-TOWLE, L. (1975) Hypoxic Sensitizers: Radiobiological Studies at the Cellular Level. *Radiology*, **117**, 453.

INGS, R. M., MCFADZEAN, J. A. & ORMEROD, W. E. (1974) The Mode of Action of Metronidazole in *Trichomonas vaginalis* and Other Micro-Organisms. *Biochem. Pharmacol.*, **23**, 1421.

MCCALLA, D. R., REUVERS, A. P. & KAISER, C. (1970) Mode of Action of Nitrofurazone. *J. Bact.*, **104**, 1126.

MOHINDRA, J. K. & RAUTH, A. M. (1976) Increased Cell Killing by Metronidazole and Nitrofurazone of Hypoxic Compared to Aerobic Mammalian Cells. *Cancer Res.*, **36**, 930.

SUTHERLAND, R. M. (1974) Selective Chemotherapy of Non-cycling cells in an *In vitro* Tumour Model. *Cancer Res.*, **34**, 3501.

THOMLINSON, R. H., DISCHE, S., GRAY, A. J. & ERRINGTON, L. M. (1976) Clinical Testing of the Radiosensitizer Ro-07-0582. III. Response of Tumours. *Clin. Radiol.*, **27**, 167.

URTASUN, R. C., CHAPMAN, J. D., BAND, P., RABIN, H., FRYER, C. & STURMWIND, J. (1975) Phase 1 Study of High Dose Metronidazole. An *In vivo* and *In vitro* Specific Radiosensitizer of Hypoxic Cells. *Radiology*, **117**, 129.

URTASUN, R. C., BAND, P., CHAPMAN, J. D., RABIN, H. R., WILLSON, A. F. & FRYER, C. G. (1976) Radiation and High Dose Metronidazole (Flagyl®) in Supratentorial Glioblastomas. *New Engl. J. Med.*, **294**, 1364.

VARGHESE, A. J., GULYAS, S. & MOHINDRA, J. K. (1976) Hypoxia Dependent Reduction of 1-(2-nitro-1-imidazolyl)-3-methoxy-2-propanol by Chinese Hamster Ovary Cells and KHT Tumour Cells *In vitro* and *In vivo*. *Cancer Res.*, **36**, 3761.